# Magnetoencephalography for epileptic focus localization based on Tucker decomposition with ripple window

**DOI:** 10.1111/cns.13643

**Published:** 2021-05-04

**Authors:** Li‐juan Shi, Bo‐xuan Wei, Lu Xu, Yi‐cong Lin, Yu‐ping Wang, Ji‐cong Zhang

**Affiliations:** ^1^ School of Biological Science and Medical Engineering Beihang University Beijing China; ^2^ Beijing Advanced Innovation Centre for Big Data‐Based Precision Medicine Beihang University Beijing China; ^3^ Beijing Advanced Innovation Centre for Biomedical Engineering Beihang University Beijing China; ^4^ Department of Neurology Xuanwu Hospital Capital Medical University Beijing China; ^5^ Brain Functional Disease and Neuromodulation of Beijing Key Laboratory Beijing China; ^6^ Hefei Innovation Research Institute Beihang University Hefei Anhui China

**Keywords:** focal epileptic, higher‐order orthogonal iteration, MEG, ripple, source imaging, tucker decomposition

## Abstract

**Aims:**

To improve the Magnetoencephalography (MEG) spatial localization precision of focal epileptic.

**Methods:**

306‐channel simulated or real clinical MEG is estimated as a lower‐dimensional tensor by Tucker decomposition based on Higher‐order orthogonal iteration (HOOI) before the inverse problem using linearly constraint minimum variance (LCMV). For simulated MEG data, the proposed method is compared with dynamic imaging of coherent sources (DICS), multiple signal classification (MUSIC), and LCMV. For clinical real MEG of 31 epileptic patients, the ripples (80–250 Hz) were detected to compare the source location precision with spikes using the proposed method or the dipole‐fitting method.

**Results:**

The experimental results showed that the positional accuracy of the proposed method was higher than that of LCMV, DICS, and MUSIC for simulation data. For clinical real MEG data, the positional accuracy of the proposed method was higher than that of dipole‐fitting regardless of whether the time window was ripple window or spike window. Also, the positional accuracy of the ripple window was higher than that of the spike window regardless of whether the source location method was the proposed method or the dipole‐fitting method. For both shallow and deep sources, the proposed method provided effective performance.

**Conclusion:**

Tucker estimation of MEG for source imaging by ripple window is a promising approach toward the presurgical evaluation of epileptics.

## Graphical abstract

The magnetic source imaging report using dipole‐fitting with spike‐window, which is inconsistent with the surgical site(right temporal lobe), while the source localization using tucker decomposition with ripple‐window, which is more consistent with the surgical site.

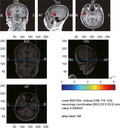

## 1 INTRODUCTION

As is known clinically, up to one‐third of patients with epilepsy are intractable.^1^ Also, drug therapy using anti‐epileptic drugs is difficult to control effectively.^2^ Epileptic foci can influence cortical excitability, which results in an abnormal discharge, not only directly in the focus or perifocally, but also in remote areas.^3^ Thus, these patients need surgical removal of epileptic foci to achieve seizure‐free.^4^ The seizure freedom after resection depends on the localization of the epileptogenic zone (EZ). However, this region is difficult to be determined due to the lack of tools that can directly measure it. High‐frequency oscillations (HFOs) are regarded as key biomarkers to find the districts of the seizure onset zones (SOZ).^5^ Accordingly, further studies are needed to validate the feasibility of the HFO window method in estimating source location.

MEG, non‐invasive and real‐time monitoring of brain function,^6^, ^7^ has been commonly used in the diagnosis of neurological diseases.^8^ Here are some reasons: First, the spatial resolution and temporal resolution are high.^9^ Second, the procedure has no harm or discomfort to patients.^10^ Third, the modern MEG has been developed very quickly and can be well used on source location with a large number of electrodes.^11^ In the presurgical evaluation of epileptics, since the neuronal activity of the patients cannot be directly measured from MEG, it is always obtained via the mathematical modeling of the inverse problem.^12^, ^13^ The precision of the spatial localization depends on the employed mathematical models.^14^, ^15^ The source location by MEG aims to explore the source activity from the sensor level. Solving the inverse problem is an underdetermined problem because the number of internal brain voxels is much more than that of external sensors.^16^, ^17^

Many algorithms were proposed to solve the ill‐posed problem in mathematical theories and other applications.^18^, ^19^, ^20^ Over the past few decades, in the field of source location by MEG or EEG, the solutions of the ill‐posed problem were under different assumptions like multiple priors, especially “sparse” priors that make more feasible the source estimation.^21^ Also, there are limitations to the source localization of distributed, bilateral, and synchronized activity.^22^ Beamforming was one of the ill‐posed solution methods used in radar systems location,^23^ sound‐source location,^24^ and brain source location.^25^ The principle of beamforming is spatial filtering, and linearly constrained minimum variance (LCMV) was the most widely used beamforming method. However, the method was sensitive to noise. To solve the problem, Tucker decomposition, a higher‐order extension of traditional singular value decomposition (SVD) and principal component analysis (PCA), can be used to retain the useful information in the signal. It has received extensive attention in image compression^26^ and multichannel signal processing.^27^

In this paper, we propose a new source imaging method for clinical preoperative assessment of surgical resection of the focal epileptic. Before source localization using LCMV, the approximate tensor of MEG is calculated by HOOI to remove the high‐frequency and low‐frequency noises. First, simulation data and clinical data were represented as multi‐way tensors. In the high dimensional space tensor, some dimensions are composed of noise, and thus, the target signal is made up of lower dimensions. The original tensor is decomposed via iteratively minimizing the difference between the estimated and original tensors. Second, the estimates were used to locate the source position by LCMV. The ripples (80–250 Hz) were then detected to compare with spikes by using the Tucker method or dipole‐fitting method. All the data, including simulation and clinical MEG data, are included in deep and shallow sources. The proposed source imaging algorithm removes the noise before the localization, remaining the most important biomarker signal fragment. Thus, the focal epilepsy focus localization is improved with the most appropriate time window.

## 2 METHODS

### 2.1 Simulation data

The simulation data are commonly used to evaluate the localization accuracy of the proposed method since it provides the spatial locations and the orientation of the dipoles. The simulation data are generated by using cosine functions, as follows:

(1) $$y\left(t\right)=A\;\text{cos}\left(2\pi ft+\varphi \right),$$

where A is amplitude, f is frequency, and φ is phase. In fieldtrip, the ft_dipolesimulation function computes the field or potential of a simulated dipole and returns a data structure identical. In this process, the amplitude, frequency, and dipole position were set as A = 1, f = 15, and φ = 10. The relative noise level was set [0:1:9]. Ten different noise levels were added to the cosine functions for evaluating the robustness of the proposed method. Above these parameters, positions are from random to specific; random positions were 300 head model positions, and specific positions were five typical positions for frontal lobe (FL), lateral temporal lobe (LTL), mesial temporal lobe (MTL), parietal lobe (PL), and occipital lobe (OL). The corresponding five coordinates were (25,66,32), (61,12,36), (29,12,36), (65,−29,88), and (60,−55,51). The length of the simulation MEG data was 600 ms. The localization accuracy was measured by the squared‐root of the spatial distance between the given ground‐truth location and the measured location.

### 2.2 Subjects

All the patients with focal epilepsy were from Xuanwu Hospital of Capital Medical University, Beijing, China. There are two main categories of patients for this analysis: (1) 19 patients whose surgical site includes the position determined by the dipole‐fitting method during the preoperative evaluation of a magnetic source imaging report in Xuanwu hospital. (2) 12 patients whose surgical site is different from the dipole‐fitting method during the preoperative evaluation. Those 31 cases include various kinds of focal epilepsy, like frontal epilepsy, lateral temporal lobe epilepsy, mesial temporal lobe epilepsy, parietal lobe epilepsy, and occipital lobe epilepsy. The subjects’ duration ranges from a few months to 30 years, and age is from 15 to 46. They are all seizure‐free after surgery over 4–6 years(Engel Ia). All the cases underwent magnetoencephalography as part of a clinical workup for epilepsy surgery for different reasons. The clinical characteristics of the subjects are shown in Tables 1 and 2.

**TABLE 1:** 19 patients whose MEG location of the magnetic source imaging report is in the surgical site

Patient ID	Sex	Age	Duration (year)	MEG reports location	Surgical site	Spike number
1	F	21	5	LPL/LTL	LTL/OL	8
2	F	24	7	RFL	RTL/Hippocampus/RFL/Insula	48
3	M	41	30	RCL	RCL/RPL	19
4	M	26	7	RTL	RTL	9
5	M	18	2	RTL/Insula	RTL	43
6	M	26	20	LFL	LFL	15
7	M	22	7	RTL	RTL/RPL	34
8	F	17	3	LFL	LFL	20
9	F	33	6	RTL/RPL	RTL	15
10	M	19	3	RTL	RTL/Hippocampus/PG	21
11	F	18	5	LTL	RTL/Hippocampus/PG	37
12	M	41	5	RTL	RTL/Hippocampus/PG	14
13	M	35	15	LTL	RTL/Hippocampus/PG	11
14	F	29	19	RTL	RTL/Hippocampus/PG	17
15	M	29	13	LTL	RTL/Hippocampus/PG	66
16	F	33	27	LTL	RTL/Hippocampus/PG	16
17	M	21	7	RTL	RTL/Hippocampus/PG	4
18	M	4	5	LPL/LTL/LOL	LPL	43
19	M	4	6	RFL	RPL	4

Abbreviations: M: male, F: female, LPL: left parietal lobe, LTL: left temporal lobe, RFL: right frontal lobe, RCL: right central area, RTL: right temporal lobe, LFL: left frontal lobe, RPL: right parietal lobe, OL: occipital lobe, and PG: parahippocampal gyrus.

**TABLE 2:** 12 patients whose MEG location of magnetic source imaging report is inconsistent with the surgical site

Patient ID	Sex	Age	Duration (year)	MEG reports location	Surgical site	Spike number
20	F	46	22	RPL	RFL	3
21	M	27	4	LPFL	LAFL	4
22	F	17	1	LIFG/Insula	LSFG	6
23	M	17	9	RIFG	RSFG	6
24	F	30	10	LMTL	LIFG	20
25	M	20	1	RMF	RSFG	3
26	F	20	1	LTL	LOL	44
27	F	33	5	LTL	LFL	76
28	F	23	8	LPL	LTL	4
29	F	15	2	LCL	LFL	42
30	M	31	10	LIFG	RTL/Hippocampus/PG	6
31	M	19	10	LIFG	LTL/Hippocampus/PG	48

Abbreviations: LPFL: left posterior frontal lobe, LAFL: left anterior frontal lobe, LIFG: left inferior frontal gyrus, LSFG: left superior frontal gyrus, RIFG: right inferior frontal gyrus, RSFG: right superior frontal gyrus, LMTL: left mesial temporal lobe, RMF: right midfrontal, LOL: left occipital lobe, and LCL: left central area.

### 2.3 Magnetoencephalography data and individual MRI

For assessing the validity and efficacy of the proposed method, simulation data are intuitive but objective. The clinical data are complicated due to different background noise varying from person to person and the sensitivity of signal to various environmental and other factors. Thus, it is extremely challenging for clinical applications. The acquisition of the MEG data during interictal of the two datasets (31 subjects) was completed in Xuanwu Hospital of Capital Medical University, Beijing, China, and the data acquisition was 1 hour long, at least 2 h later after a seizure, with 1000 points per second (the timepoints were: [0:0.001:3600]) for 60 min by the Elekta Neuromag, during which they keep resting‐state with eyes closed.

Dataset 1: the MEG signal of 19 patients whose MEG location of the magnetic source imaging report is in the surgical site. Dataset 2: the MEG signal of 12 patients whose MEG location of the magnetic source imaging report is inconsistent with the surgical site. The MEG signals were collected continuously in 102 positions. There were three sensors at each position: one magnetometer and two gradiometers. Thus, 306 channels of the MEG signal were obtained. The 60‐min MEG signal was divided into six segments for computational convenience.

In the source location process, an individual head model offers advantages over the standard head model. The individual MRI was obtained by the 3.0 T SIEMENS magnetic resonance imaging devices of Xuanwu Hospital of Capital Medical University. 192 coronal MRI sections of the patients were acquired to remodel the head shape by the software Mricron.

### 2.4 Spikes

For the person with epilepsy, MEG spikes were defined with more than 2 vs <1 of the 3 experienced clinical neurophysiologists based on the average montage with an analog bandwidth of 0.1~70 Hz and a notch filter of 50 Hz in the MEG center of Xuanwu Hospital of Capital Medical University. The spike numbers of every patient are shown in Tables 1 and 2, where the spike label is given as a time point. In source localization, the spike window 250 ms was used: 100 ms before the time point and 150 ms after the time point.

### 2.5 Source localization algorithm

**FIGURE 1:**
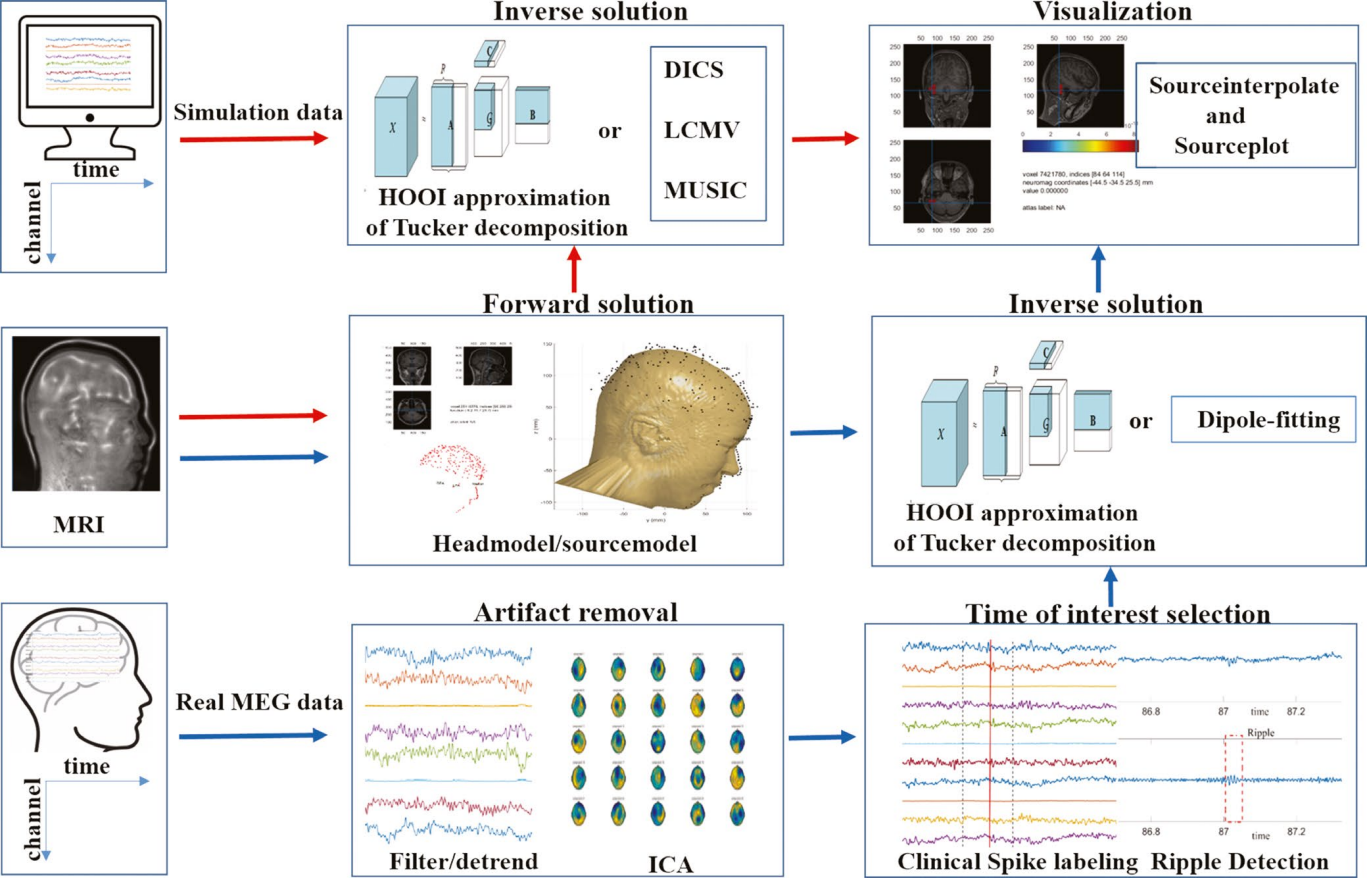
The source localization flowchart. The red arrows: the flowchart of simulation data, including the forward solution, the inverse solution problem (Tucker, DICS, LCMV, and MUSIC), and visualization. The blue arrows: the flowchart of real MEG data, including artifact removal, time of interest selection (spike and ripple), the forward solution, the inverse solution problem (Tucker, dipole‐fitting), and visualization

The flowchart of the source localization is described in Figure 1. The overall process consisted of five parts: artifact removal, forward problem, time of interest selection, inverse problem (HOOI approximation of MEG), and visualization.

#### 2.5.1 Data preprocessing

In the data preprocessing step, the power frequency, linear trend, and higher‐order trend are removed from MEG data. Then, independent component analysis (ICA) is applied for removing ECG. Also, we use a semi‐automatic way to make a visual selection of the data for efficient preprocessing. Some functions in fieldtrip like ft_rejectvisual are used to reject channels containing artifacts using a summary view of channels transformed into z‐scores by variance analysis.

#### 2.5.2 Forward problem

The channel position, head model, and source model were registered in a coordination system with the same units. Here, the coordinate system, neuromag, is adopted. Also, individual MRI is used to prepare the head model by constructing a surface description and volume conductor model of the brain. Single shell is used as the head model, where the head shape from the sensor level is presented via 306 channels MEG signals, and then the anatomical MRI is spatially aligned with head coordinates based on external fiducials or anatomical landmarks by rotation, scaling, and translation. The forward solution can be computed through this process when the head model, channel positions, and source are given.

#### 2.5.3 Ripple detection

Ripples, 80–250 Hz, are defined as at least four oscillations standing out from the background. In order to save time and effort, an automated method, root mean square (RMS), was used to determine ripples, after band pass of 80–250 Hz, the scores of ripple power should exceed [3 9], and the duration of a ripple should last to at least 15 ms. It was completed in matlab programs. From the clinical experience, it was found that the waveform of ripples is often had amplitude modulation, that is, rise gradually and then descend slowly. So for the detention candidate ripples, the false‐positive ripples caused by noise were removed manually by an experienced clinical neurophysiologist after the automated method.

#### 2.5.4 HOOI approximation of MEG

In the proposed method, the Tucker decomposition based on higher‐order orthogonal iteration (HOOI) is used to estimate the ranks (R_1_, R_2_, … R_N_) of the N‐order input tensor MEG. The output tensor is denoted as T. The Tucker decomposition is formulated as follows:

(2) $$\left[\;\left[G;U^{\left(1\right)},U^{\left(2\right)},\cdots U^{\left(N\right)}\right]\;\right]\overset{\mathrm{def}}{=}G\times_{1}U^{\left(1\right)}\times_{2}U^{\left(2\right)}\cdots \times_{N}U^{\left(N\right)},$$

where G is the core tensor containing the main information, and $U^{\left(i\right)}$ are factor matrices of the original tensor.

HOOI is an efficient method to calculate the core tensor and the factor matrices, where singular value decomposition (SVD) replaces the eigenvalue decomposition. The computation is conducted in an iterative process as follows:

1. The high‐order SVD (HOSVD) is used to compute factor matrices: *k* = 0, $G^{\left(0\right)}$ = zero tensor (all the entries are 0).
2. Given *K* = *k* + 1, *n* = 1, 2, … *N*, (3) is computed:

​

(3) $$B^{\left(k\right)}\leftarrow X\times_{1}U^{(1)T}\cdots \times_{n‐1}U^{(n‐1)T}\times_{n+1}U^{(n+1)T}\times_{N}U^{\mathrm{NT}}.$$

The mode‐n unfolding of $B^{\left(k\right)}$ is computed, and the number of major singular values $R_{n}$ is determined as follows:

(4) $$U^{\left(N\right)}\leftarrow U\left(:,1:R_{n}\right).$$

- The k^th^ core tensor is computed until it convergence:

​

(5) $$∥G^{\left(k\right)}‐{G^{\left(k‐1\right)}∥}_{F}<\varepsilon .$$

- The core tensor and the factor matrices are output.

​

The pseudo‐code of the proposed algorithm is described in Algorithm 1.

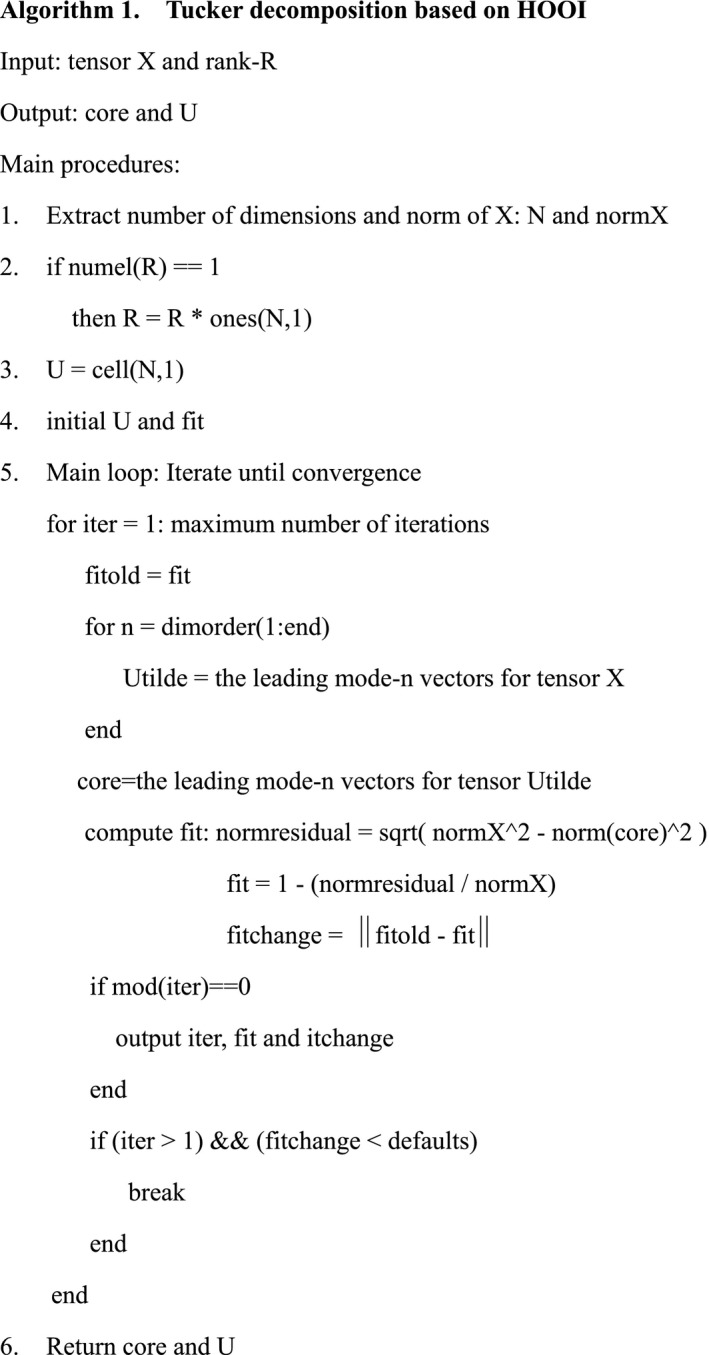

#### 2.5.5 Inverse problem and source display

Beamformer dipole analysis is used for the inverse problem to compute the covariance matrix based on the approximation data of the MEG data; then, the source is displayed. The beamformer principle is as follows: signals are filtered by spatial, temporal, and frequency domain information to obtain the information of a specified direction while attenuating the noise interference from other directions. As the beamformer method, LCMV is used in the time domain. The key idea of the method is to guarantee a certain gain in the target signal direction in space and minimize the power output of the MEG array. LCMV produces a 3D spatial distribution of the power of the neuronal sources.

For simulation data, the proposed Tucker decomposition is compared with LCMV, DICS, and MUSIC. On the other hand, for the real clinical MEG data, the proposed Tucker decomposition is compared with the clinically used dipole‐fitting method in terms of the effectiveness of the source localization of ripples with spikes. The flowchart of the comparison is shown in Figure 1, where the red and blue arrows represent the simulation data module and real clinical MEG data, respectively.

### 2.6 Statistical analysis

The statistical analyses were performed utilizing Origin software v2018. To analyze potential relationships between the new Tucker method and dipole‐fitting method with spike window or ripple window, hypothesis testing, the paired t tests were used. In all statistical analyses conducted, a *p* < 0.05 was viewed as statistically significant.

## 3 RESULTS

### 3.1 Result of simulation data

**FIGURE 2:**
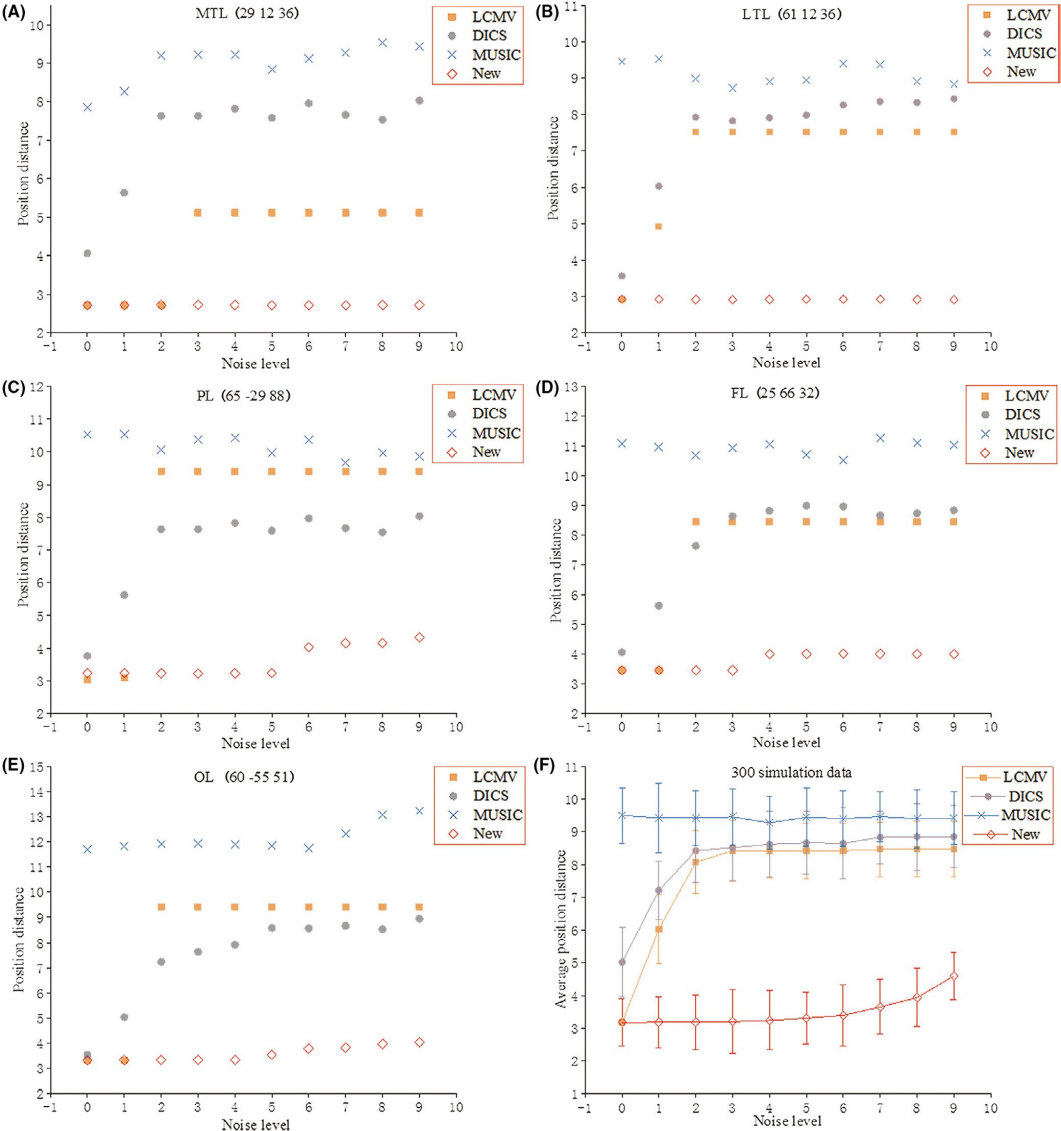
(A‐E) The position distances of the five typical positions in the simulation data. F: average position distance of the 300 random positions in the simulation data

The coordinates of the five typical positions are as follows: (29 12 36) for MTL, (61 12 36) for LTL, (65 −29 88) for PL, (25 66 32) for FL, and (60 −55 51) for OL. Note that (29 12 36) is a deep position. The position distances of the estimated location by LCMV, DICS, MUSIC, and the proposed method (New) are shown in Figure 2. The proposed method provides higher accuracy over the compared method for MTL, especially for higher noise levels (>3), showing its effectiveness for the deep source (Figure 2A). The proposed method also outperforms the compared methods in terms of localization accuracy for the shallow sources, LTL and PL (Figure 2B and C). Further, as shown in Figure 2D and E, the position distances of the proposed method are shorter than the compared methods for the FL and OL. The proposed method also shows higher localization accuracy for the 300 random positions (Figure 2F). When a noise level is higher than 5, the position distance of the proposed method increases but still shorter than the compared methods, LCMV, DICS, and MUSIC.

### 3.2 Result of ripple detection and spike acquisition

**FIGURE 3:**
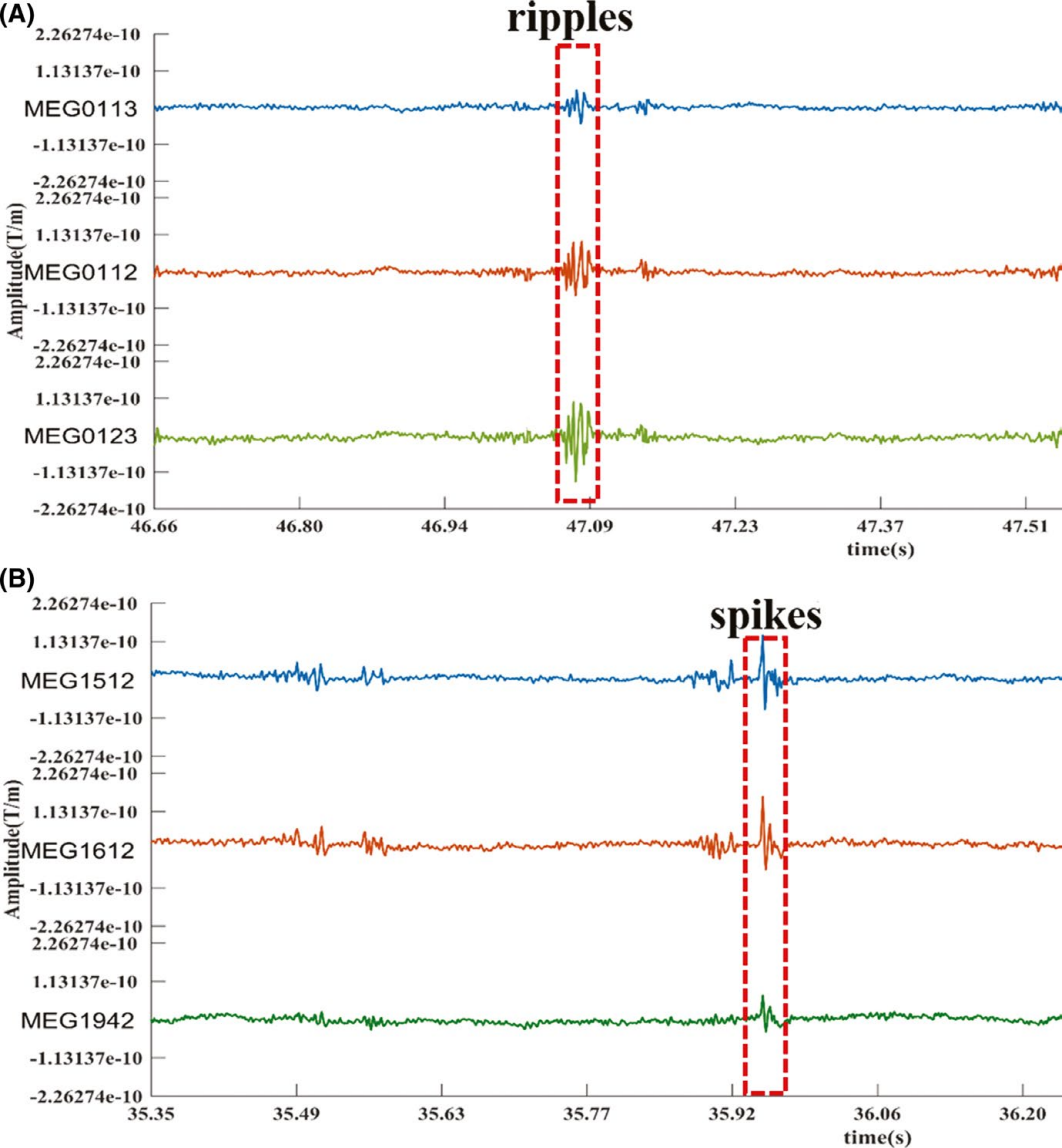
Representative image of MEG ripples(3A) and spikes(3B)

The two datasets (31 patients) were used to evaluate the effectiveness of the proposed Tucker decomposition with ripple. All of the candidate ripples were automatically detected by the RMS after bandpass filtered (80–250 Hz). From clinical experience, the positive ripples were gradient that was slowly rose and then declined. In contrast, some ripples are followed after visible noise and were considered negative ripples. Negative ripples were removed from the candidate ripples. A Final result of ripple detection was shown in Figure 3A.

For spikes, based on the time points annotated by clinical neurophysiologists, an spike example of MEG was shown in Figure 3B.

### 3.3 Result of clinical MEG datasets

**FIGURE 4:**
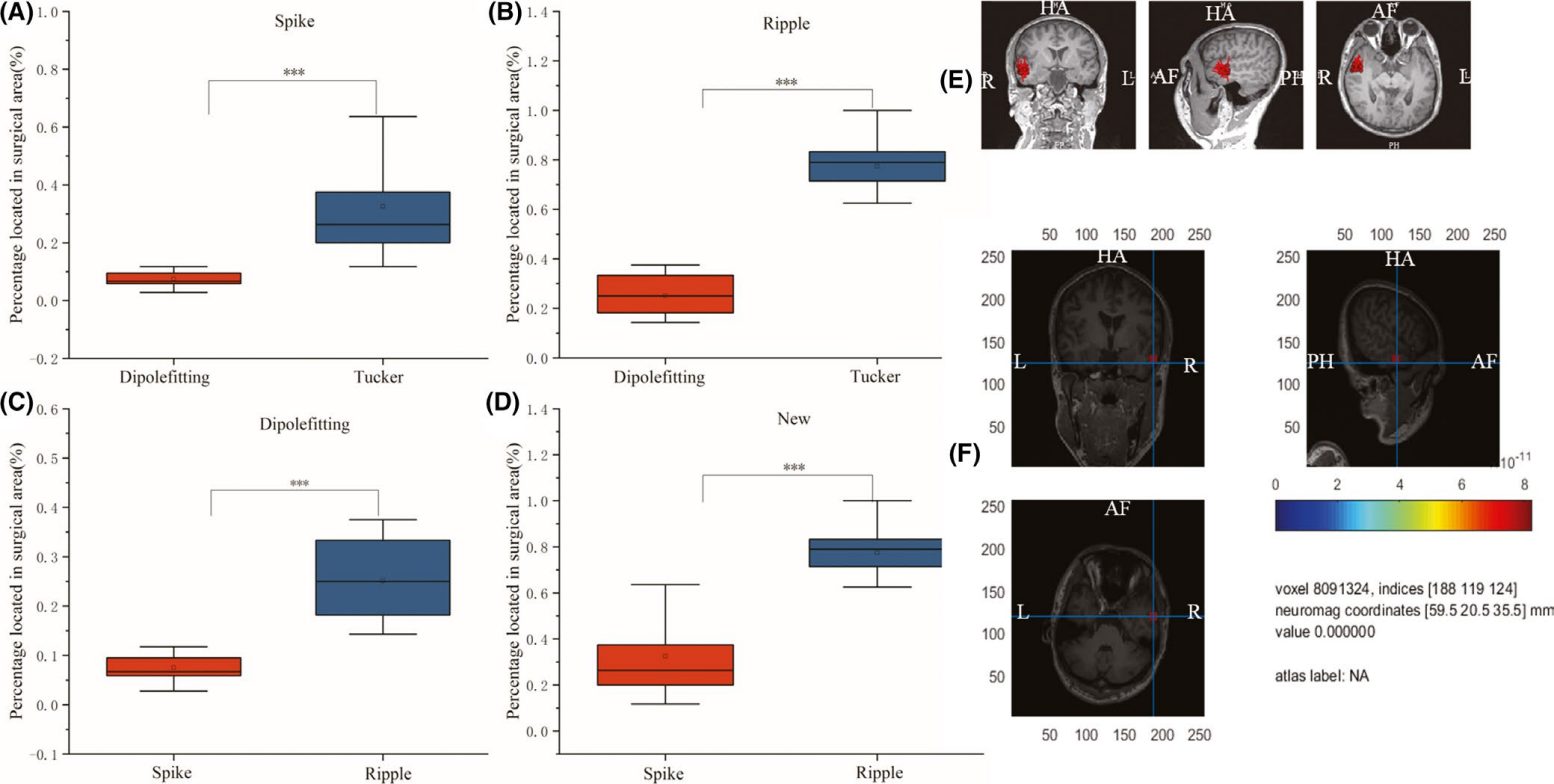
Results on dataset 1. (A) the percentage of spikes located in the surgical area, (B) the percentage of ripples located in the surgical area, (C) the percentage of spikes and ripples obtained by the dipole‐fitting method, and (D) the percentage of spikes and ripples obtained by the proposed Tucker decomposition method. Significant differences (***p < 0.001) were observed for the proposed Tucker method with dipole‐fitting method by using spike window or ripple window. E and F are examples of results on dataset 1. (E) the magnetic source imaging report using dipole‐fitting with spike window, F: the source localization using tucker decomposition with ripple window

Figure 4 shows the results on dataset 1. The percentages of spikes and ripples located in the surgical area are depicted for each patient, which are obtained with the proposed Tucker decomposition and dipole‐fitting. As shown in Figure 4A, the proposed Tucker decomposition method localizes spikes (>0.2) more than the dipole‐fitting method (<0.2) (*p *< 0.001). As shown in Figure 4B, the proposed Tucker decomposition method localizes ripples (>0.6) more than the dipole‐fitting method (<0.4) (*p *< 0.001). As is shown in Figure 4C, ripples (>0.15) have a higher location accuracy than that of spikes (<0.15) for the dipole‐fitting method (*p *< 0.001). As is shown in Figure 4D, ripples (>0.6) have a higher location accuracy than that of spikes (<0.4) for the dipole‐fitting method (*p *< 0.001).

Figure 4E and F show an example for dataset 1, the magnetic source imaging report using spike by dipole‐fitting from the MEG central (Figure 4E) and the localized source of Tucker decomposition using ripple (Figure 4F). It is shown that the source location result of the proposed method using a ripple window is consistent with that of dipole‐fitting using a spike window. Note that both are all consistent with the surgical site: right temporal lobe.

**FIGURE 5:**
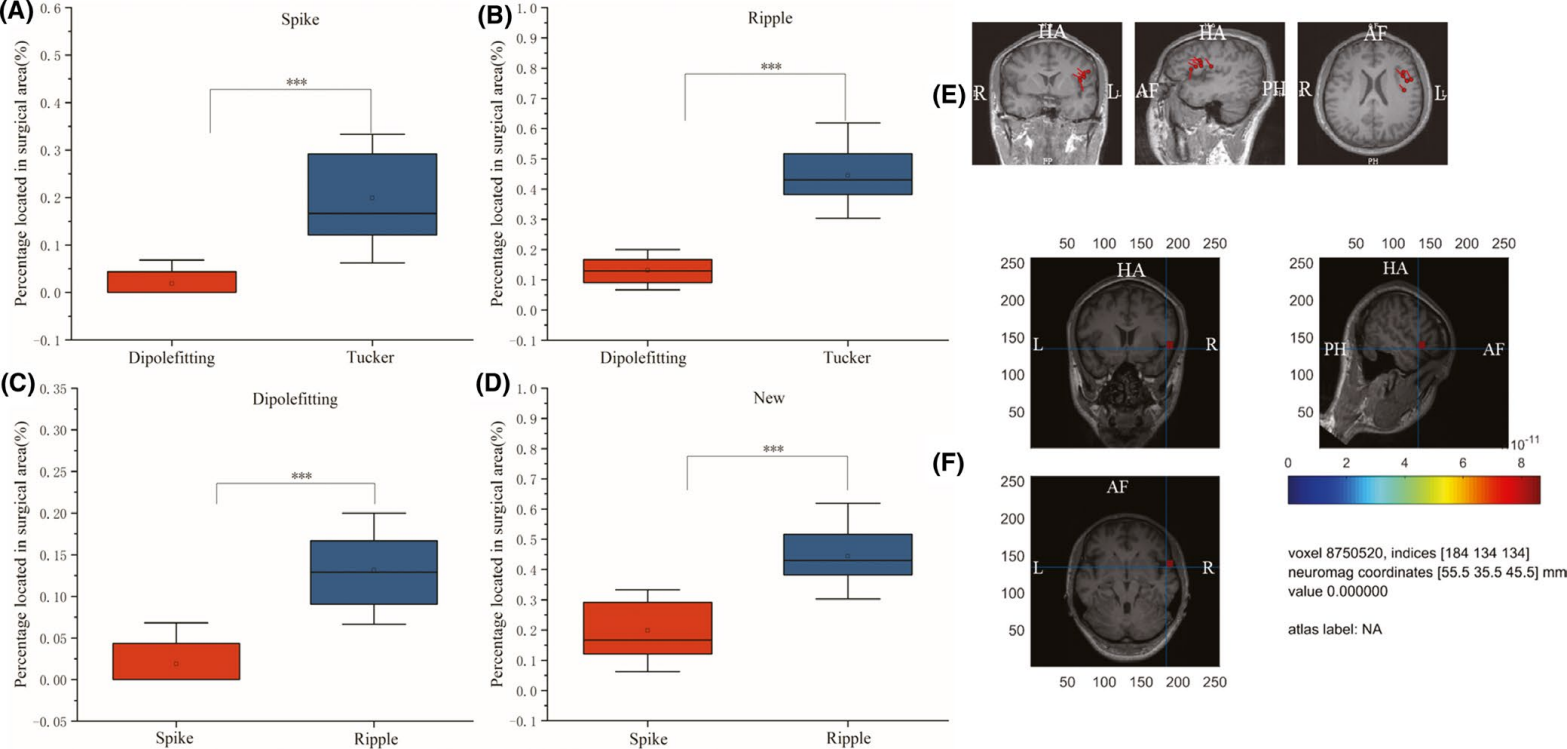
Results on dataset 2. (A) the percentage of spikes located in the surgical area, (B) the percentage of ripples located in the surgical area, (C) the percentage of spikes and ripples obtained by the dipole‐fitting method, and (D) the percentage of spikes and ripples obtained by the proposed Tucker decomposition method. Significant differences (***p < 0.001) were observed for the proposed Tucker method with dipole‐fitting method by using spike window or ripple window. E and F are examples of results on dataset 2. (E) the magnetic source imaging report using dipole‐fitting with spike window, (F) the source localization using tucker decomposition with ripple window

Figure 5 shows the results on dataset 2. Similar to dataset 1, the percentages of spikes and ripples located in the surgical area are depicted for each patient, which are obtained with the proposed Tucker decomposition and dipole‐fitting. The trend is the same as the results on dataset 2. As shown in Figure 5A, the proposed Tucker decomposition method (>0.1) localizes spikes more than the dipole‐fitting method (<0.1) (*p *< 0.001). As shown in Figure 5B, the proposed Tucker decomposition method(>0.3) localizes ripples more than the dipole‐fitting method (<0.2) (*p *< 0.001). As is shown in Figure 5C and D, ripples have a higher location accuracy than that of spikes for both the proposed method and the dipole‐fitting method (*p *< 0.001).

Figure 5E and F show an example for dataset 2, where the magnetic source imaging report using spike by the dipole‐fitting of MEG central (Figure 5E)and the localized source of Tucker decomposition using ripple (Figure 5F) are depicted. It is shown in Figure 5F that the source location result of the proposed method using ripple window is consistent with that of the surgical site: right temporal lobe, contrary to F, E shows that source location using spike window by dipole‐fitting is inconsistent with the surgical site.

**FIGURE 6:**
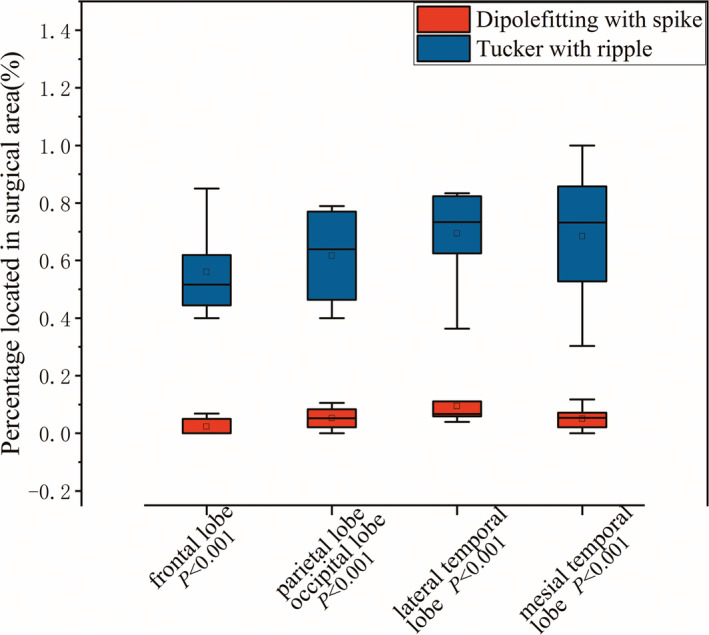
An individualized calculation of accuracy for five types of 29 epilepsy patients (frontal lobe, lateral temporal lobe, mesial temporal lobe, parietal lobe, and occipital lobe). Significant differences (***p < 0.001) were observed for frontal lobe, lateral temporal lobe, mesial temporal lobe patients, and the percentage located in surgical area of Tucker by ripple window is also higher than that of dipole‐fitting with spike for the three parietal lobe and one occipital lobe patients(***p < 0.001)

In practical clinics, dipole‐fitting with ripple time window has been a widely recognized source location method. However, as shown in Figure 6, for the enrolled 5 types of all 31 patients(frontal lobe, lateral temporal lobe, mesial temporal lobe, parietal lobe, and occipital lobe), the Tucker decomposition with ripple window has a promising advantage over the dipole‐fitting method (the *p* < 0.001 of frontal lobe, lateral temporal lobe, and mesial temporal lobe; for parietal lobe and occipital lobe, the patient number is too small in clinic to make a statistical analysis, so 3 parietal lobe patients and 1 occipital lobe patients were analyzed together (*p *< 0.001)) indicating that the proposed Tucker method with ripple window outperforms the dipole‐fitting with spike window.

## 4 DISCUSSION

MEG measures the brain's magnetic fields that are quantifiable at various scalp sites, where the magnetic field is generated by the electric field. The main contributor to the scalp MEG is the neuronal discharge in different parts of the brain. MEG is a more powerful technology used to identify epileptogenic zones noninvasively than EEGs, as EEG has a weak signal‐to‐noise ratio. The high spatial resolution is important to study brain functional disease, while MEG offers a higher spatial resolution than EEG, which allows the source of neuronal activity to be more accurately located.

In this study, we propose a source localization algorithm based on MEG to find biomarkers of brain activity. In the proposed source localization method, MEG is considered to be a tensor, and the factor matrix and the core tensor were calculated through orthogonal iteration. The proposed tensor model is reconstructed from its constituent parts. The effect of HOOI makes an orthogonal constraint on Tucker to assess the uniqueness of the solution. High‐frequency and low‐frequency noises are removed simultaneously, and computational complexity decreases with a rank reduction.^28^

The proposed method was first evaluated in the simulation data. The proposed method outperforms the compared methods: LCMV, DICS, and MUSIC in terms of the localization accuracy, especially for signals with a high signal‐to‐noise ratio (SNR). So MEG with high SNR has an advantage over EEG. The simulation position encompasses several sources, including a deep source and four shallow sources. The proposed method achieved higher localization accuracy up to 2. The proposed method has a certain breakthrough significance for deep source epilepsy other than the shallow source. For clinical epilepsy MEG data, the percentage of spikes and ripples located in the surgical area obtained by the proposed method is higher than that of clinical dipole‐fitting. Note that the real MEG data also included deep sources and shallow sources, and thus, it is useful for different kinds of epilepsy. The results show that the proposed method can make great advances in source location accuracy for deep sources in clinical.

There were many studies on HFO for focal epilepsy^29^, ^30^ to find the neurons responsible for generating seizures, that is, for identifying the EZ. While many HFOs are detected from EEG,^31^, ^32^ EEG signals are at coarse spatial resolutions compared with MEG. MEG is a useful technology to detect interictal epileptic discharges (IEDs) (ie, spikes, spike and wave discharges, or sharp waves) or HFO noninvasively. Thus, the HFO of MEG demonstrates considerable promise for preoperative evaluation of epilepsy.^33^

The interictal HFOs of MEG are useful in defining the spatial extent of the seizure onset zones. Considering the important role of HFO in the preoperative evaluation of epilepsy, we combined the Tucker decomposition with ripple. The performance of the combination was compared with different combinations: Tucker and dipole‐fitting using spike window and ripple window. The MEG data of 31 epilepsy patients were used as the validation dataset. The results show that the localization accuracy of the Tucker method is higher than the dipole‐fitting method for both ripple and spike windows. When combined ripple with Tucker decomposition, the positioning accuracy was the best among different combinations. We conclude that the localization results using Tucker decomposition before LCMV can remove redundant noise so that the results become more accurate than dipole‐fitting. Combining with the ripple window provides more accurate results than that of the spike window due to the predictive information about upcoming seizures or seizure onset zone (SOZ) carried by the time window, which also guides iEEG to localize epileptogenic zones.

Accordingly, the proposed method with the ripple window can be an excellent tool for MEG source localization in the preoperative evaluation of epilepsy. Still, this study has some limitations. First, the MEG recording is expensive, and thus the algorithm verification requires a relatively long time. In addition, a single source and few sources were hypothesized when validating the method on simulation data and the real MEG data. Future works will include the explorations for multiple sources. At last, the number of different types patients should be increased in order to make a better statistical analysis, especially parietal lobe and occipital lobe patients, which are rare relatively.

## 5 CONCLUSION

In this work, we propose a new source localization method for MEG. A reduced rank of a tensor is employed to remove redundant information, which is contrary to precise positioning. The experimental results on simulation data and clinical MEG data show that the Tucker method with ripple window is more effective than clinical dipole‐fitting with spike window. These results lay the foundation for the vital role of ripple in source localization in the preoperative assessment of epilepsy surgery.

## CONFLICTS OF INTEREST

None of the authors have any financial disclosure or Conflicts of interest.

## Supporting information

Supplementary Material

## Data Availability

Research data are not shared due to privacy or ethical restrictions.
