# Supplementary figures and images for: Magnetoencephalography for epileptic focus localization based on Tucker decomposition with ripple window
Source: CNS Neurosci Ther. 2021 May 4;27(7):820–30. doi: 10.1111/cns.13643 (PMC8193700; doi:10.1111/cns.13643)

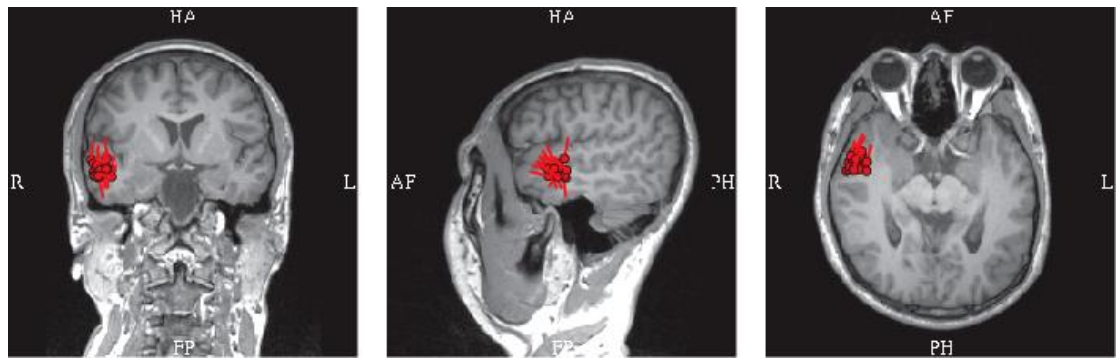

Full unedited image for Figure 4E

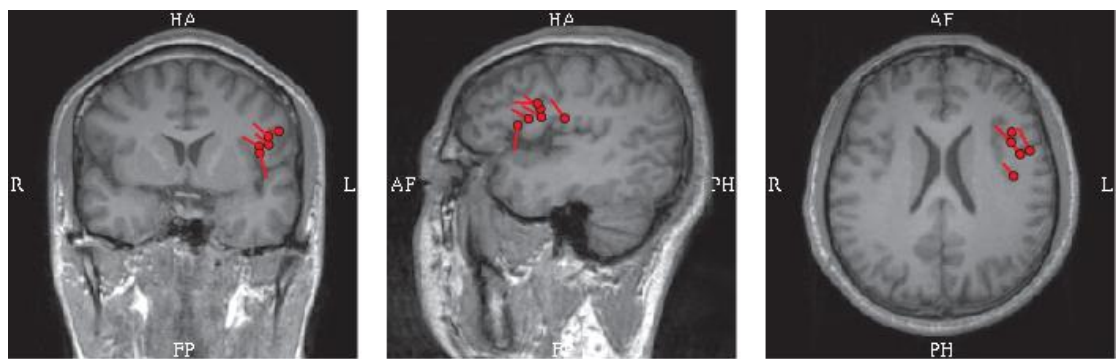

Full unedited image for Figure 5E

Supplement: Supplementary file 1 — Supplementary Material [file CNS-27-820-s001.pdf]
